# Synergistic acupuncture and neuromodulation for chronic insomnia: a structured narrative review with systematic search and future directions

**DOI:** 10.3389/fneur.2025.1663585

**Published:** 2025-09-11

**Authors:** Wang Meng, Wang Mingqiang, Zhang Junyang, Peng Bin, Wu Qiong, Wang Chao

**Affiliations:** ^1^Institute of Basic Theory of Chinese Medicine, China Academy of Chinese Medical Sciences, Beijing, China; ^2^College of Traditional Chinese Medicine, Jiangxi University of Traditional Chinese Medicine, Nanchang, China

**Keywords:** chronic insomnia, acupuncture, neuromodulation, autonomic nervous system, corticolimbic functional connectivity, synergistic therapy, nonpharmacological intervention

## Abstract

Insomnia represents a significant global public health issue, affecting approximately 10% of adults with chronic symptoms and up to 20% with intermittent episodes. Closely associated with chronic diseases such as depression, anxiety, and cardiovascular conditions, insomnia markedly impairs patients’quality of life and imposes substantial economic burdens. Current treatments include pharmacological and non-pharmacological approaches. Pharmacotherapy primarily involves benzodiazepines, effective in short-term symptom relief but associated with long-term risks such as dependency, tolerance, and cognitive impairment. Recently introduced dual orexin receptor antagonists offer improved safety profiles but lack sufficient clinical evidence and remain costly. Melatonin and its receptor agonists have contentious efficacy, while antihistamines are discouraged for chronic use due to adverse effects. Herbal therapies have limited high-quality evidence to support routine clinical use. Among non-pharmacological treatments, cognitive behavioral therapy for insomnia (CBT-I) is recognized for clear efficacy, yet patient adherence and availability of trained specialists remain problematic. Exercise interventions, bright light therapy, and music therapy show preliminary positive effects; however, inconsistencies in intervention parameters and methodological quality necessitate further research. Recent advances highlight the potential of acupuncture and neuromodulation technologies, including transcranial magnetic stimulation (TMS), transcranial direct current stimulation (tDCS), and vagus nerve stimulation (VNS). Acupuncture effectively improves sleep through modulation of autonomic nervous function, endocrine regulation, and remodeling of sleep-related neural circuits, demonstrating sustained benefits and high safety. Neuromodulation techniques offer rapid onset and precise targeting. Preliminary evidence indicates that combining acupuncture and neuromodulation techniques could synergistically enhance treatment efficacy, efficiency, and personalization. However, existing studies remain limited. Large-scale, multicenter randomized controlled trials are needed to clarify efficacy, safety, and to optimize clinical protocols for these integrative approaches. We conducted a structured narrative review with a systematic search (2010–2025), including 93 studies for qualitative synthesis; evidence certainty was summarized using four qualitative categories (High/Moderate/Low/Very low).

## 1 Introduction

Insomnia is a significant global health concern affecting approximately 10% of adults chronically, with up to 20% experiencing occasional insomnia symptoms (1). Recent studies have demonstrated that insomnia not only adversely impacts patients’ daily lives but is also closely associated with various chronic conditions, including depression, anxiety, and cardiovascular diseases (2–4). Moreover, insomnia imposes substantial economic burdens on healthcare systems; in the United States alone, the direct and indirect costs associated with insomnia exceed 100 billion USD annually (5). Current clinical guidelines recommend pharmacological interventions and cognitive behavioral therapy for insomnia (CBT-I) as the primary treatments. However, both therapeutic approaches have notable limitations, such as the potential for dependency and high relapse rates associated with pharmacological treatments, and low patient adherence and difficulty maintaining efficacy for CBT-I (6).

Conventional pharmacological therapies, although clearly effective in rapidly alleviating insomnia symptoms, commonly lead to dependence, drug tolerance, and noticeable side effects, including headaches and memory impairment, when used long-term (7, 8). CBT-I, an important non-pharmacological therapy with demonstrated clinical efficacy (9), is heavily dependent on patient engagement and requires ongoing follow-up and lengthy treatment cycles (6). Although recognized as a first-line intervention in various clinical guidelines, CBT-I has not been widely implemented as a mainstream insomnia therapy in many regions due to economic constraints and poor patient adherence (10, 11). With the increased use of electronic devices such as smartphones (12) and rising life stress (2), the incidence of insomnia is currently trending upward. Therefore, exploring therapeutic methods that effectively avoid or mitigate these limitations is crucial for improving clinical treatment outcomes and alleviating patient suffering.

In recent years, with the deepening research on insomnia mechanisms and therapeutic interventions, acupuncture and modern neuromodulation techniques have demonstrated certain beneficial effects in improving insomnia (13–15). Some exploratory studies have attempted to combine acupuncture with neuromodulation techniques, aiming to provide multidimensional therapeutic outcomes and compensate for the limitations of individual therapies (16, 17). However, it is important to acknowledge that research into these integrative approaches remains limited and is still in the preliminary exploration phase.

Based on the above background, this article systematically reviews and evaluates comprehensive treatment methods for insomnia, with a particular emphasis on the potential clinical value of combining acupuncture with modern neuromodulation techniques such as transcranial magnetic stimulation (TMS), transcranial direct current stimulation (tDCS), and vagus nerve stimulation (VNS), aiming to provide references and insights for developing more effective, safer, and personalized therapeutic strategies in the future.

## 2 Materials and methods

### 2.1 Literature search and selection

This work is a structured narrative review with a systematic literature search (2010–2025); quantitative meta-analysis was not performed. A systematic literature search was performed on June 30, 2025, in PubMed (*n* = 753), Web of Science (*n* = 866), and the Cochrane Library (*n* = 296) to identify studies published between January 1, 2010 and June 30, 2025. We combined three concept blocks in title/abstract fields:

Insomnia terms: “insomnia” OR “chronic insomnia” OR “primary insomnia” OR “sleep disturbance” OR “sleep disorder” OR “sleep quality” OR PSQI.

Intervention terms: “acupuncture” OR “electroacupuncture” OR “auricular acupuncture” OR “scalp acupuncture” OR TMS OR “transcranial magnetic stimulation” OR rTMS OR tDCS OR tACS OR VNS OR tVNS OR taVNS OR neuromodulation.

Mechanistic terms: “Heart rate variability (HRV) “OR cortisol OR melatonin OR “Gamma-aminobutyric acid (GABA)” OR “functional connectivity (fMRI/EEG)” OR plasticity OR “hypothalamic–pituitary–adrenal(HPA) axis”.

The initial search returned a total of 1,915 records; following deduplication, 1,650 unique entries remained. Two independent reviewers then screened titles and abstracts, excluding 1,557 records that did not meet pre-specified inclusion criteria, and assessed the full text of 93 articles—all of which were included in the final analysis. Any discrepancies were resolved by consensus.

References 18-40—covering benzodiazepines, dual orexin receptor antagonists, melatonin, and antihistamines—were identified separately via manual citation tracking of authoritative clinical practice guidelines and targeted PubMed searches; these were not part of the primary systematic search. Supplementary citations (e.g., 45, 46, 102) that discuss key neurophysiological mechanisms or the clinical relevance of acupuncture/neuromodulation were also manually retrieved to provide foundational mechanistic context.

### 2.2 Inclusion and exclusion criteria

Studies were selected according to the following criteria to ensure methodological rigor and thematic focus on behavioral therapies, acupuncture, and neuromodulation for chronic insomnia.

#### 2.2.1 Inclusion criteria

Population: Adult patients (≥18 years) diagnosed with chronic insomnia by DSM-5 or ICSD-3 criteria.

##### 2.2.1.1 Interventions (at least one)

Behavioral/Cognitive Therapies: CBT-I and its components (sleep hygiene, sleep restriction, stimulus control, relaxation training, cognitive restructuring), exercise interventions (e.g., Tai Chi), bright light therapy, music therapy.

Acupuncture Modalities: Manual acupuncture, electroacupuncture, auricular acupuncture, scalp acupuncture, acupoint embedding.

Neuromodulation Techniques: Repetitive/transcranial magnetic stimulation (rTMS/TMS), transcranial direct current stimulation (tDCS), transcranial alternating current stimulation (tACS), transcutaneous or auricular vagus nerve stimulation (tVNS/taVNS), or combined protocols.

Comparators: Sham or placebo interventions, usual care, active controls (e.g., CBT-I alone), or no-treatment controls.

##### 2.2.1.2 Outcomes

Clinical efficacy: PSQI, sleep latency, total sleep time, sleep efficiency, and adverse events.

Mechanistic endpoints: HRV, serum cortisol or melatonin levels, GABA concentration, fMRI/EEG, HPA-axis markers, and neural plasticity indicators.

##### 2.2.1.3 Study design

Randomized controlled trials and nonrandomized clinical trials.

Systematic reviews and meta-analyses that (a) report a reproducible search strategy and (b) focus on RCTs of behavioral, acupuncture, or neuromodulation interventions for insomnia. (c) Mechanistic studies conducted in chronic insomnia patients or validated insomnia animal models.

Publication Date: January 1, 2010 to June 30, 2025, with priority given to studies published from January 1, 2020 onward.

Language: English full-text publications.

#### 2.2.2 Exclusion criteria

We excluded case reports, narrative reviews lacking a defined search strategy, study protocols, letters, and conference abstracts without full data. Basic science studies were excluded unless they were conducted in validated insomnia animal models with clear translational endpoints directly mapping to clinical phenotypes (e.g., sleep latency/efficiency, HPA-axis markers); such studies, when retained, were used solely to contextualize mechanisms and were not used to grade clinical efficacy (and were rated as low certainty in our qualitative framework).

Pediatric populations (<18 years), non-insomnia sleep disorders, mixed cohorts without separable insomnia data, and non-English publications were excluded.

### 2.3 Evidence appraisal and certainty rating

Two reviewers qualitatively appraised study limitations across standard domains (randomization/allocation, blinding, missing data, outcome measurement, and selective reporting) and summarized the certainty of evidence for key outcomes using four categories—high, moderate, low, and very low. This was a qualitative synthesis; no formal domain-level tool scoring or quantitative meta-analysis was performed. Data extraction focused on study design, sample size, intervention parameters, and the direction and magnitude of primary sleep outcomes as reported (e.g., Pittsburgh Sleep Quality Index (PSQI) changes and *p* values). In practice, the included literature mapped to the Moderate or Low categories; no topic achieved High certainty (owing to small sample sizes, heterogeneity, and imprecision), and none met our threshold for Very low certainty (because evidence was primarily clinical rather than indirect or severely limited).

## 3 Conventional treatments for chronic insomnia

### 3.1 Pharmacological treatments

The primary categories of medications currently used for insomnia treatment each have distinct advantages and disadvantages:

#### 3.1.1 Benzodiazepines and benzodiazepine receptor agonists

Common benzodiazepines include diazepam and lorazepam, while benzodiazepine receptor agonists include zolpidem, zaleplon, and eszopiclone. These medications are characterized by rapid onset, effectively addressing difficulty initiating sleep and nighttime awakenings in the short term (18, 19). However, studies have shown that long-term use can lead to drug tolerance, dependence, and withdrawal symptoms (20). Additionally, these medications may impair cognitive functions (21, 22), cause nighttime confusion, and increase the risk of falls (23, 24). Consequently, most guidelines recommend their use only for short durations and advocate strict control over treatment duration (6, 25). Some scholars even suggest avoiding these medications altogether due to their significant adverse effects (26).

#### 3.1.2 Low-dose sedating antidepressants

Commonly used medications such as trazodone and low-dose doxepin can improve sleep quality and alleviate mild emotional disturbances, making them suitable for insomnia patients with comorbid emotional disorders (27, 28). However, their effectiveness exhibits considerable individual variation, and they can cause adverse effects such as falls (29), urinary retention, and dry mouth (28).

#### 3.1.3 Dual orexin receptor antagonists

Representative medications include suvorexant, lemborexant, and daridorexant. DORA improves sleep by inhibiting orexin A/B neuropeptides, thus reducing excessive central wakefulness (30). Overall, this drug category is associated with lower dependence and better safety profiles (31). The most commonly reported side effects include somnolence, nasopharyngitis, and headache (32). Drawbacks of these medications include high treatment costs and limited current clinical data; consequently, only some drugs within this category are recommended by clinical guidelines for treating insomnia (33, 34).

#### 3.1.4 Melatonin/melatonin receptor agonists

Melatonin and its receptor agonists (such as ramelteon) regulate circadian rhythms and have been widely used to treat insomnia, particularly in patients aged 55 years and older. Nevertheless, recent literature reviews have produced conflicting conclusions regarding the effectiveness of melatonin and its receptor agonists (35–38). Thus, clinical guidelines frequently exhibit ambiguity regarding their recommendation for routine use in treating insomnia (25).

#### 3.1.5 Antihistamines

Antihistamines commonly used for insomnia treatment are typically over-the-counter medications such as diphenhydramine and doxylamine. Despite clinical use, these drugs are generally not recommended due to potential side effects and possible drug tolerance (39). Recent studies have also suggested an association between these medications and increased mortality, warranting caution in their use (40).

#### 3.1.6 Herbal/botanical therapies

Although some reviews have summarized the use of herbal and botanical therapies for insomnia relief (41, 42), there is a relative lack of randomized controlled trial data. While these therapies are practiced clinically in certain countries and regions, guidelines do not recommend herbal and botanical remedies as standard treatments for insomnia (6).

Overall, although some commonly used medications in clinical practice can rapidly alleviate insomnia symptoms, issues such as cognitive impairment, impaired consciousness, and insufficient clinical evidence leading to medication misuse remain significant concerns. These issues highlight the fact that while pharmacological treatments are among the most widely utilized methods, numerous factors may adversely impact patient treatment outcomes and overall health during their clinical application.

### 3.2 Non-pharmacological treatments

#### 3.2.1 Cognitive behavioral therapy for insomnia

CBT-I is a structured, evidence-based, non-pharmacological therapy currently recommended as a first-line intervention for chronic insomnia (43). It primarily works by altering unhealthy sleep behaviors and modifying negative cognitive patterns, thereby helping patients establish effective sleep patterns and improving sleep quality (44). CBT-I comprises several key components, including psychoeducation and sleep hygiene (SH), relaxation therapy (RT), sleep restriction therapy (SRT), stimulus control therapy (SCT), and cognitive therapy (CT).

##### 3.2.1.1 Psychoeducation and SH

Psychoeducation is frequently utilized as an adjunct therapy for various psychological disorders (45, 46), mainly serving educational and advisory roles (45). In insomnia treatment, psychoeducation typically includes basic information regarding the role and functions of sleep, age-related changes, and the circadian rhythm regulation, exemplified by the classical two-process model of sleep regulation (47, 48). In the practical application of CBT-I, psychoeducation is generally integrated across therapeutic components rather than presented independently. Sleep hygiene is a central component of CBT-I. Although the academic community has not reached a consensus on the precise definition of SH, it is broadly considered a set of behavioral and environmental recommendations aimed at promoting healthy sleep (49). These recommendations typically include regular exercise, noise reduction during sleep, and maintaining consistent sleep schedules (50). The efficacy of SH was once questioned (51), but with accumulating evidence, recent research acknowledges SH as having a positive impact on sleep outcomes (52, 53). Overall, the lack of consensus on the precise components of SH complicates the establishment of universally quantifiable and objective standards in clinical practice (49). Although some studies advocate personalized SH interventions tailored to different populations (54, 55), large-scale research remains limited.

##### 3.2.1.2 RT

RT employs various relaxation techniques, such as progressive muscle relaxation, deep breathing exercises, meditation, or guided imagery, to help patients reduce physical and mental tension and alleviate anxiety, thereby facilitating sleep onset (56). Progressive muscle relaxation is widely applied; Mehdi Harorani et al. (57), for example, employed progressive muscle relaxation therapy and found significant improvements in anxiety and sleep quality among burn patients in the intervention group. This therapy also positively impacts insomnia in patients with hip fractures (58). Karuna Datta et al. demonstrated through clinical experiments that yoga nidra practice improves N3 sleep, total wake time, and subjective sleep quality among patients with insomnia (59). Despite its frequent clinical application, some meta-analyses suggest that RT may have limited overall effectiveness or even counterproductive outcomes (60).

##### 3.2.1.3 SRT

SRT aims to increase sleep drive by initially restricting the patient’s “time in bed” to their average total sleep duration, thereby promoting more concentrated and continuous sleep. Initially, patients schedule their bedtime based on actual sleep duration, gradually increasing time spent in bed as sleep efficiency improves, ultimately enhancing sleep quality (61). SRT has a long history of clinical use and is relatively mature, demonstrating positive short-term effects on various insomnia severity indicators such as sleep latency and sleep efficiency (62). Recent experimental studies have also confirmed good long-term efficacy and flexible implementation through telephone-guided interventions (63). However, deliberate sleep deprivation during initial treatment stages often leads to adverse effects including extreme fatigue, daytime sleepiness, headaches, mood fluctuations, decreased energy, and reduced motivation (64). These side effects may negatively influence patient adherence and thus compromise intervention outcomes (65).

##### 3.2.1.4 SCT

SCT involves altering patients’associations with the bed and bedroom, reestablishing a positive connection between bed and sleep. Specific measures include going to bed only when sleepy and, if unable to fall asleep within 20 min, leaving the bed to engage in relaxing activities until feeling sleepy again (66). Clinical trials indicate that SCT effectively alleviates insomnia symptoms and reduces pre-sleep cognitive activation (67). However, the overall quality of existing experimental studies on SCT is relatively low, and further rigorous research is necessary to clearly evaluate its efficacy and underlying mechanisms (68).

##### 3.2.1.5 CT

CT focuses on identifying and challenging irrational beliefs and negative thoughts related to sleep, such as “I will definitely sleep poorly” or “Not getting enough sleep will affect tomorrow,” employing cognitive restructuring to reduce sleep-related anxiety and excessive worry (60). Clinical trials exclusively employing CT have been relatively infrequent in recent years. Rikard Sunnhed et al. (69), however, found that internet-delivered CT demonstrated favorable efficacy as a standalone therapy for insomnia.

In summary, CBT-I, as a preferred non-pharmacological treatment for insomnia, effectively improves sleep quality without pharmacological side effects and offers good long-term benefits with some of its components (63). It also positively influences patients with comorbid psychiatric conditions (9). Nonetheless, CBT-I presents certain limitations: SH, as a component, lacks consensus (49) and has even been used as a placebo in randomized controlled trials (70); RT may potentially exacerbate insomnia symptoms (60); and SRT might impair daytime energy (64). Furthermore, CBT-I requires prolonged treatment periods, high patient motivation, and sustained adherence, all of which substantially impact therapeutic efficacy (65). Limited availability of standardized CBT-I services due to a shortage of qualified therapists further constrains treatment accessibility in certain regions (71). Therefore, although CBT-I is a first-line therapy for insomnia, further clinical validation and refinement are necessary, alongside the development of novel therapeutic methods.

#### 3.2.2 Exercise, bright light therapy, and music therapy

##### 3.2.2.1 Exercise interventions

EI enhances deep sleep regulation by increasing biosynthesis of melatonin precursors in the brain (72). Preliminary studies suggest exercise may also improve sleep–wake rhythms by modulating hypothalamic–pituitary–adrenal axis activity (73). Clinical interventions typically involve running or stepping exercises (72); recent studies indicate Tai Chi can also alleviate insomnia (74). Additional research (75) found that incorporating exercise into CBT-I can help sustain cognitive therapy effectiveness.

However, EI presents limitations including substantial variability in exercise prescriptions (intensity, frequency, timing), inadequate adherence assessment, and insufficient individualized recommendations for varying age groups and comorbidities (76, 77). Despite supportive evidence, high-quality research remains insufficient.

##### 3.2.2.2 Bright light therapy

BLT involves exposure to intense light during morning or evening to suppress delayed melatonin secretion and reset the circadian rhythm regulated by the suprachiasmatic nucleus, reducing sleep latency and enhancing sleep efficiency (78, 79). Qin Wang et al. (78), through a randomized controlled trial, showed that daily 30-min morning exposures to 7,500-lux white light for 2 weeks improved PSQI from 12.4 ± 3.1 to 7.2 ± 2.8 (*Δ* = −5.2; *p* < 0.001). Another trial (80) using 10,000-lux morning exposure for 30 min/day over 2 weeks improved sleep efficiency and reduced daytime sleepiness, fatigue, and mood disturbance (between-group *p* < 0.05).

Despite accumulating evidence supporting BLT’s efficacy, significant heterogeneity in intervention parameters—light intensity (2,000–10,000 lux), wavelengths (white vs. blue), exposure duration (15–60 min), and timing (morning vs. evening)—hinders high-quality meta-analyses, greatly affecting comparability and clinical implementation (81). Other research suggests moderate intensity (900–6,000 lux) and longer duration (≥1 h) nighttime exposure is more effective for extending total sleep time, though efficacy varies across parameter combinations (82). Consequently, standardized BLT protocols require further development.

##### 3.2.2.3 Music therapy

MT involves pre-sleep listening to slow-paced, soothing music to activate parasympathetic responses, reduce cortisol and anxiety levels, alleviate tension, and promote sleep (83). Helle Nystrup Lund et al. (84) found significant improvements in PSQI scores after 4 weeks of MT in patients with insomnia and depression (from 14.1 ± 3.2 to 8.3 ± 2.5, *p* < 0.001), shorter sleep latency (*p* < 0.01), and enhanced subjective well-being compared to controls. Li Chang et al. (85) demonstrated that MT combined with aerobic exercise significantly improved PSQI scores compared to controls (by 5.3 points, *p* < 0.001), benefiting multiple sleep dimensions. However, significant variability in music type (classical vs. nature sounds), duration (15–60 min), delivery methods (headphones vs. speakers), and individual music preferences (86, 87), challenges in blinding, reliance on subjective evaluations, and lack of objective physiological metrics (e.g., polysomnography) limit robust conclusions (88). Additionally, some studies suggest limited evidence for MT’s sleep-improving effects (89).

## 4 From single therapies to synergistic strategies: acupuncture + neuromodulation

### 4.1 Acupuncture

Acupuncture, an essential component of Traditional Chinese Medicine (TCM), involves the insertion of needles at specific acupoints (e.g., Shenmen [HT7], Baihui [GV20]), accompanied by manual or electrical stimulation, to unblock meridians and balance Yin and Yang (90). However, recent studies indicate that its clinical application is not guided solely by TCM theory; anatomical and other biomedical theories are also widely utilized. For example, acupuncture can restore autonomic and endocrine homeostasis (91, 92). Currently, acupuncture is primarily regarded as a therapeutic modality rather than a distinct medical discipline. Numerous empirical studies support acupuncture’s efficacy in treating insomnia (93). Wang et al. (94) demonstrated acupuncture’s effectiveness in alleviating insomnia symptoms, observing enhanced efficacy with appropriate acupoint combinations. Weng et al. (95), through systematic review and meta-analysis, concluded that acupuncture significantly improves PSQI scores in breast cancer patients experiencing insomnia. Besides traditional acupuncture, Yin et al. (96) applied electroacupuncture (EA) at TCM-specific points for 8 weeks, significantly reducing PSQI scores from 16.1 ± 3.5 at baseline to 9.9 ± 2.7 post-treatment, outperforming both sham EA (11.0 ± 3.0) and controls (13.5 ± 3.2) (both *p* < 0.001), with lasting effects. Other acupuncture-derived techniques or combined approaches also yield promising results for insomnia; for instance, Lu et al. (97) found moderate evidence for the effectiveness of acupoint embedding therapy and auricular acupuncture combined with traditional acupuncture, although additional robust evidence is needed.

Current mechanistic studies of acupuncture for insomnia primarily focus on three areas. First, acupuncture modulates autonomic function by enhancing vagal tone and increasing HRV. Meira do Valle et al. (98) suggested acupuncture effectively reduces sympathetic stress, possibly by activating the vagus nerve, thereby increasing HRV and coherence. Li et al. (99) reviewed two decades of literature, consistently identifying the autonomic nervous system as a primary acupuncture target. Second, acupuncture exerts endocrine regulatory effects, reducing serum cortisol and promoting melatonin secretion rhythms. Li et al. (100) observed significant improvements in sleep quality and reduced daytime fatigue in insomnia patients, with elevated plasma melatonin and reduced cortisol levels, attenuating HPA axis hyperactivation. Huang et al. (101), utilizing the traditional TCM “Ziwu Liuzhu” acupuncture method, noted significantly higher melatonin levels in acupuncture-treated rats compared to medication-treated counterparts. Third, acupuncture reshapes central neural networks, directly impacting sleep-related cortical–limbic circuits. Wang et al. (94), using resting-state functional magnetic resonance imaging (RS-fMRI), reported enhanced prefrontal-hippocampal connectivity and increased electroencephalographic slow-wave amplitude following multi-acupoint manual acupuncture compared to sham. Jin et al. (102) further confirmed enhanced functional connectivity between default mode network and cognitive control network structures following manual stimulation at Zusanli (ST36) in healthy subjects (*p* < 0.01). Collectively, these studies indicate acupuncture’s cortical–limbic network remodeling capacity significantly enhances slow-wave sleep generation and maintenance.

Acupuncture exhibits several notable advantages for insomnia treatment. Firstly, as a non-pharmacological therapy, it eliminates medication dependence (103) or central adverse effects (104), thus offering high safety. Secondly, acupuncture’s therapeutic benefits have proven durability, with multiple clinical trials demonstrating effects persisting several months after treatment completion (96, 105). Lastly, acupuncture synergistically integrates with therapies such as CBT-I (106), exercise interventions (107), and neuromodulation (16), enhancing overall treatment efficacy. However, acupuncture has limitations, including lengthy treatment courses with associated discomfort (94), potentially affecting patient adherence and tolerance. Additionally, lack of standardized consensus regarding acupoint selection, needle insertion depth, retention duration, and stimulation intensity introduces considerable variability, complicating efficacy comparisons and reproducibility (108).

### 4.2 Neuromodulation techniques

Neurostimulation techniques—transcranial magnetic stimulation (TMS), transcranial direct current stimulation (tDCS), transcranial alternating current stimulation (tACS), and vagus nerve stimulation (VNS)—constitute an emerging class of non-pharmacological interventions that modulate central or peripheral nervous system activity to rebalance network excitation and inhibition (109). Collins et al. (110) reported that rTMS treatment significantly improved mood and sleep quality in patients with severe depression and comorbid insomnia, independently of age and medication factors, accompanied by notable reductions in PSQI scores. Sun et al. (111), through systematic analysis of previous studies, concluded that rTMS is a safe and effective treatment for insomnia, significantly improving PSQI scores whether applied as primary or adjunctive therapy.

Transcranial electrical stimulation primarily encompasses tDCS and tACS, with increasing clinical trials conducted in recent years. Bakhshayesh Eghbali et al. (112) demonstrated through clinical trials that active tDCS significantly improved sleep quality in patients experiencing insomnia after traumatic brain injury, exhibiting greater reductions in PSQI scores compared to sham tDCS. Additionally, tDCS has shown efficacy in improving sleep quality among patients with depression (113). Regarding tACS, Wang et al. (114, 115) validated its ability to significantly reduce PSQI scores, shorten sleep latency, and increase total sleep duration. Other researchers (116), applying alpha-frequency tACS stimulation over the medial parietal cortex for chronic insomnia, reported notably enhanced and sustained improvements in PSQI scores and sleep quality.

Clinical studies of VNS currently include transcutaneous vagus nerve stimulation (tVNS) and transcutaneous auricular vagus nerve stimulation (taVNS). Zhang et al. (117) reported positive effects of tVNS on sleep quality improvement in patients with insomnia induced by high-altitude conditions. Zhang et al. (118) demonstrated that taVNS significantly reduced the severity of insomnia symptoms, with sustained effects lasting beyond 20 weeks. Current research on neuromodulation techniques continues to expand, including the exploration of combined neuromodulation methods. For instance, Zhou et al. (119) found that combining tDCS and rTMS achieved significant therapeutic benefits within 2 weeks of treatment, with sustained efficacy observed in subsequent follow-up periods. Some researchers (120) have proposed the potential clinical value of combining taVNS with slow-paced breathing exercises for treating insomnia.

The mechanisms underlying neuromodulation primarily involve three aspects. First is autonomic regulation, predominantly via modulation of vagus nerve function. Butt et al. (121), through detailed anatomical and neural tracing studies, demonstrated that taVNS directly activates vagal afferent branches located in the auricular concha, transmitting signals to the nucleus tractus solitarius and vagal nuclei within the brainstem, thus enhancing parasympathetic tone and improving heart rate variability (HRV). Similarly, tDCS enhances parasympathetic function and HRV via cortical-brainstem pathways (122), whereas rTMS may improve control over cardiovascular autonomic regulation by modulating functional connectivity between the left dorsolateral prefrontal cortex (DLPFC) and central autonomic networks (123). Second is endocrine modulation; rTMS treatment for insomnia was shown to significantly reduce serum cortisol, adrenocorticotropic hormone (ACTH), high-sensitivity thyroid-stimulating hormone, and free T3/T4 levels alongside improvements in insomnia symptoms. This modulation likely involves the DLPFC’s regulatory influence over the hypothalamic–pituitary–adrenal (HPA) and hypothalamic–pituitary-thyroid axes (124). Furthermore, other studies suggest rTMS alleviates insomnia symptoms through elevated GABA levels (125). The third aspect involves modulation of network synchronization and neural plasticity. For example, taVNS may enhance functional connectivity between the insula and medial prefrontal cortex, improving the dynamic balance between interoceptive awareness and cognitive experiences. This regulatory effect potentially affects mind–body interactions, elucidating taVNS’s therapeutic mechanism (126). Additional studies (121) found unilateral tVNS increased the negative compatibility effect, a GABA-related behavioral marker, promoting regional neural plasticity changes. Similarly, tDCS facilitates sleep by activating glutamatergic projections from the infralimbic cortex (IL) to the ventrolateral preoptic nucleus (VLPO) (127).

Neuromodulation technologies demonstrate clear advantages in insomnia treatment. Firstly, these methods exhibit high safety and tolerability; numerous randomized controlled trials involving insomnia or related populations have reported only minor, transient adverse effects, such as mild headaches or local discomfort, without serious adverse events (115, 117, 119). Secondly, treatments such as rTMS often achieve rapid improvements in sleep quality and architecture within a short period (111). Finally, neuromodulation devices offer adjustable parameters and precise targeting capabilities. For example, tDCS devices feature adjustable current intensity, polarity configurations, and multi-array electrodes capable of precisely stimulating critical sleep–wake centers such as frontoparietal or dorsolateral prefrontal cortices. However, mechanistic understanding of neuromodulation remains limited, with some experiments still in preliminary stages (128). Furthermore, certain researchers suggest that the efficacy of neuromodulation techniques requires further exploration; some studies (129) indicate tACS does not show significantly greater therapeutic effects compared to sham treatments for insomnia symptoms. Overall, Krone et al. (115), in a systematic review, concluded that neuromodulation therapies warrant additional rigorous verification.

#### 4.2.1 Comparative overview

To facilitate direct comparison across major neuromodulation approaches in chronic insomnia, Table 1 summarizes key attributes of rTMS, tDCS/tACS, and tVNS/taVNS, including their proposed mechanisms, stimulation targets and parameters, typical clinical efficacy (PSQI change), common adverse events, and overall evidence level.

**Table 1:** Comparative summary of neuromodulation techniques in chronic insomnia (ΔPSQI indicates mean change where reported).

Technique	Mechanism	Targets and parameters	ΔPSQI (mean)	Certainty (qualitative)	References
rTMS	Modulation of prefrontal–limbic connectivity; HPA axis regulation	Left DLPFC; 5–20 Hz protocols (e.g., 10 Hz, 1,200 pulses/session)	−4.0 to −6.1	Moderate	(110, 111)
tDCS	Cortical excitability enhancement; autonomic balance; cortisol reduction	DLPFC; 1–2 mA, 20 min/session (various montages)	−3.8 to −4.2	Moderate	(112, 113)
tACS (*α*-frequency)	Oscillatory entrainment; slow-wave augmentation	Medial parietal cortex; 10 Hz, 1–2 mA, 30 min/session	−4.7 to −5.0	Moderate	(114, 116)
Vagus nerve stimulation (tVNS/taVNS)	Vagal-afferent activation; ↑ parasympathetic tone; HPA axis suppression	Auricular concha/tragus; 20–25 Hz, ~200–250 μs pulse width	−3.9 to −4.5	Moderate	(117, 118)
tDCS + rTMS	Synergistic excitability + network remodeling	tDCS (2 mA over DLPFC) + rTMS (10 Hz, 1,200 pulses)	−6.0	Moderate	(119)
Systematic review	Composite mechanisms across modalities	Various protocols	N/A	Low	(115)

Certainty categories (High/Moderate/Low/Very low) reflect a qualitative synthesis based on study design/size and consistency across studies. Certainty ratings are qualitative and summarize evidentiary strength by design/size and cross-study consistency; they are not per-outcome meta-analytic estimates and do not imply pooled effect sizes or confidence intervals. In this review, ratings fell into the Moderate or Low categories; High and Very low were not assigned.ΔPSQI values are study-specific and reflect within-study changes over heterogeneous durations; they are not directly comparable across modalities.

### 4.3 Synergistic strategy: acupuncture + neuromodulation

Recent trials have begun to demonstrate the clinical benefit of combining acupuncture with neuromodulation. Zhang et al. (17) compared low-frequency rTMS alone to rTMS plus manual acupuncture in patients with chronic insomnia and observed a significantly greater reduction in PSQI score in the combination group (*p* < 0.05). More recently, Zhou et al. (119) reported that a two-week adjunctive protocol of tDCS plus rTMS produced faster and more durable improvements in sleep efficiency than either modality alone, with superiority maintained at four-week follow-up.

These results provide empirical support for a synergistic clinical effect. However, to date there are no published studies directly probing the combined neurophysiological and autonomic mechanisms of acupuncture and neuromodulation. Therefore, the following hypotheses remain highly exploratory and require empirical validation in future trials.

Accordingly, drawing upon the mechanistic insights presented in Sections 3.1 and 3.2, together with findings from aforementioned RCTs, we propose two specific, testable hypotheses:

(i) Vagal-adrenal priming of infralimbic to ventrolateral preoptic (IL-VLPO) plasticity:

Acupuncture-induced enhancement of vagal-adrenal axis activity may create a permissive neurochemical environment (e.g., increased GABA (125), reduced cortisol (100)) that amplifies tDCS-mediated synaptic plasticity within the IL-VLPO sleep-promoting pathway.

(ii) Prefrontal-limbic recalibration with HPA-axis suppression:

rTMS-driven remodeling of dorsolateral prefrontal–limbic connectivity may synergize with acupuncture-mediated suppression of HPA-axis hyperactivity to more effectively attenuate the chronic stress responses underpinning insomnia (124).

These hypotheses remain speculative and await direct empirical testing through rigorous experimental and clinical studies in future. Well-powered, factorial-designed trials—incorporating multimodal neuroimaging (fMRI, EEG), autonomic (HRV), and endocrine (cortisol, melatonin) biomarkers—are needed to test differential effects across insomnia subtypes.

### 4.4 Limitations and prospects

Preliminary studies demonstrate combined acupuncture-neuromodulation efficacy (16, 17), but related research remains nascent. Considering rising insomnia prevalence, limited treatment options, and inadequate CBT-I availability, alternative therapies merit exploration (130). However, combined therapies present complexity in training, implementation costs, and the current paucity of large-scale randomized controlled trials (RCTs). Future studies should employ interdisciplinary teams (acupuncturists plus neuromodulation operators), large-sample multi-center randomized double-blind trials, objective-subjective endpoints, and prolonged follow-up to validate safety and efficacy comprehensively.

### 4.5 Safety, adverse effects, and contraindications

Although the preceding sections have emphasized the potential benefits of acupuncture and neuromodulation techniques in chronic insomnia, the safety profiles and possible adverse events of these interventions also warrant thorough discussion. Clinical studies have reported that rTMS treatment is often accompanied by transient headaches or scalp discomfort (131); moreover, a very small number of patients may face a risk of seizure (132), necessitating cautious evaluation or avoidance in individuals with a prior history of epilepsy or severe brain injury. The adverse effects of tDCS/tACS typically include mild tingling or erythema at the stimulation site; adverse events were predominantly mild and transient, and serious events were rare in included trials (133). Similarly, tVNS/taVNS procedures should be performed under monitored conditions in individuals with high-risk arrhythmias or severe cardiopulmonary insufficiency (134). By contrast, acupuncture, as a low-risk non-pharmacological therapy, primarily causes minor local adverse events such as bruising, infection, or needling discomfort; however, the incidence rate is extremely low, and serious adverse events are rare and mostly related to improper operation (135, 136). Future research should incorporate multi-center, large-sample randomized controlled trials with concurrent monitoring of adverse events and safety outcomes to establish a more comprehensive and balanced risk–benefit assessment framework.

## 5 Conclusion and future directions

Despite diverse treatments, significant gaps in current sleep physiology and insomnia pathophysiology models remain (115). The complementary integration of acupuncture and neuromodulation provides an innovative, personalized therapeutic approach, warranting further standardized, multi-center RCTs to optimize procedures, confirm sustained efficacy and safety, and explore differential responses across insomnia subtypes.
